# E-concussion? an investigation of the representation of head impact events and concussion within popular sport-based video games

**DOI:** 10.1371/journal.pone.0328627

**Published:** 2025-07-22

**Authors:** Freja J. Petrie, Nathan Howarth, Samantha C. Bureau, Chris J. Nowinski, James S. Woodward, Isaac Lockett

**Affiliations:** 1 Assistive Technology Innovation Centre (ATiC), University of Wales Trinity St David, Swansea, Wales; 2 University of Exeter Medical School, University of Exeter St Luke’s Campus, Exeter, England; 3 Concussion Legacy Foundation Canada, Toronto, Canada; 4 School of Sport and Exercise Sciences, Faculty of Health, Liverpool John Moores University, Liverpool, England; 5 Sport and Exercise Sciences Research Institute, Ulster University, Belfast, Ireland; 6 School of Life Sciences, Pharmacy and Chemistry, Faculty of Health, Science, Social Care and Education, Kingston University, London, England; King Faisal University, SAUDI ARABIA

## Abstract

Guidelines have been produced to support the management of concussion, an injury commonly experienced in amateur and elite sports. To improve adherence to concussion guidelines, there is a need to improve their dissemination to the public. Mass media is inherently well positioned to distribute this information; however, concussion must be framed accurately and appropriately to avoid the spread of misinformation. To date the extent to which concussion is depicted in video games is unknown, despite their widespread audience and evidenced use as educational tools. Therefore, this study investigated the representation of concussion (and head impact events) in four sport-based video games. Matches from EA Sports FC 24 (EA Sports, n = 16), EA Sports FC 25 (EA Sports, n = 16), Rugby League Live 4 (Tru Blu, n = 18) and Rugby 22 (Nacon and Bigben Interactive S.A, n = 18) were simulated via a Playstation 5 (Sony Interactive Entertainments). Frequency of direct head impact events and references to concussion were reported. Head impact events were observed in all games (EA Sports FC 24 n = 253, EA Sports FC 25 n = 331, Rugby League Live 4 n = 93, Rugby 22 n = 215). A single concussion was reported in Rugby League Live 4 via a dialogue box. No references to medical intervention or concussion protocols were made in any games analysed. The lack of reference to concussion protocols is a missed opportunity to exemplify appropriate concussion behaviours.

## Introduction

Industry forecasts project that the video game market will reach a value of £516.61 billion by 2029, with the number of active users predicted to rise to three billion [1]. In parallel with this growth, recent literature has explored the educational potential of video games [2,3] and the relationship between Sport-based Video Games (SBVGs) and real-world sporting events [4–6]. Notably, video games are increasingly recognised as educational tools that enhance engagement with physical activity and promote positive lifestyle choices [6,7] while offering an accessible and enjoyable medium through which individuals can participate in sport [4,5,8]. In light of these developments, SBVGs designed to provide realistic representations of sporting experiences may offer a novel opportunity to model appropriate concussion behaviours, aiding concussion recognition.

Concussion can occur when impulsive forces are transmitted to the brain [9]. This transmission of forces may result from a direct blow to the head (commonly referred to as a Head Impact Event, HIE [10]) or from an indirect blow to the body [9]. The term Head Acceleration Event (HAE) encompasses both types of force transmission to the head, irrespective of whether a concussion diagnosis is confirmed [11]. When players exhibit signs of suspected concussion, sporting governing bodies and national health authorities advise that established concussion protocols are followed to minimise the risk of further injury [12–14]. These guidelines are widely available online, written in accessible language, and are frequently linked to free online courses offering more detailed information. Despite the availability of this information, access to and engagement with concussion education remains inconsistent. Some professional athletes demonstrated *“moderate”* knowledge of concussion and *“good”* knowledge of concussion symptoms [15], but this is contrasted by reports that 74% of Jordanian, 24% of Irish, and 9% of American collegiate athletes had never received concussion information [16], while 65% of American and 42% of Japanese high school students had not accessed any concussion education [17,18].

In many cases, the onus is on the individual to actively seek out concussion information. In the absence of structured delivery of education, concussion-related knowledge may instead be acquired through passive exposure to mass media. The importance of passive media in disseminating health information can be examined through the Media System Dependency Model [19], highlighting the public’s increasing reliance on media to understand complex social issues. To apply this model within a sporting context, it could be posited that individuals increasingly depend on media coverage to track developments in sport and interpret emerging concerns about athlete wellbeing. Consequently, the media’s role in shaping public understanding of concussion and appropriate care practices has grown in significance. For example, a survey of parents of student-athletes (n = 1083) found that parent’s primary sources of concussion information were local news (31%), national news (24%), and sport-specific media (24%), with most respondents perceiving these sources as trustworthy [20]. However, the credibility assigned to such media may be misplaced. Media coverage of concussion often lacks clinical precision, employs narrative-driven reporting, or editorial decisions that prioritise drama over accuracy [21–23]. A case study examining media reports of a goalkeeper’s concussion during the 2018 Champions League Final revealed that 38% of articles either downplayed or misrepresented the injury [24]. Similarly, an analysis of online rugby articles found that the term *“head knock”* was used in 29% of cases to describe concussion [25], illustrating the persistence of colloquial and minimising language.

To date, the portrayal of concussion within SBVGs has not been examined. This gap is noteworthy given the potential of video games, like other forms of media, to influence public perceptions of health and safety issues [26,27]. Drawing on Cultivation Theory and Social Learning Theory [28,29], researchers have argued that media representations can normalise risky behaviours, particularly amongst younger audiences [30–32]. For example, many of the world’s most popular video games omit basic safety precautions, such as helmet or seatbelt use, and depict injury events without realistic consequences or protective responses [33]. Similarly, examination of how mental illness is portrayed in video games highlights a broader pattern of problematic health depictions, including violent stereotypes and exaggerated narratives [34].Within this context, an analysis of how concussion is represented in SBVGs is essential to evaluate whether these games reinforce safe norms or promote unhealthy behaviours. Broader media effects frameworks, including Cultivation theory [35,36] and the Entertainment-Education model [37], underscore the role of realism in shaping player’s understanding of health issues. This raises broader ethical and social considerations. As technology advancements enable increased realism, there is an argument that injuries could warrant depiction based on their capacity to impact real-life outcomes. Should this be the case, the ethical responsibility of the video game industry to engage with health issues becomes a topic of importance, particularly given heightened awareness around player safety in both professional and youth sport [38].

The educational potential of SBVGs has been explored from multiple perspectives, with research noting their capacity to engage learners and encourage self-reflection. In secondary school settings, an ethnographic investigation highlighted how students became immersed in the gameplay, often identifying with virtual athletes by referring to them in the first person: *“I’m playing”*, *“I am* (example team)*”* [39]. The authors highlighted that this immersivity underscored the educational potential of SBVGs as students could make choices in an extended reality and reflect on the resulting actions of these choices [39]. In more experimental contexts, the playing of SBVGs has been shown to improve knowledge of sport-specific rules and procedures. Experimental groups who played *“Madden NFL”* (American football) or *“Don Bradman Cricket 14”* had significantly greater knowledge of field layout and player positioning and the rules and terminology, respectively [40,41]. Whilst examined primarily with marketing implications in mind, in-game adverts were shown to improve brand awareness further illustrating the educational potential of these games [42]. This realism contributes positively to user enjoyment, which in turn prolongs engagement with these games, and therefore learning [43].

An understanding of how video games currently represent concussion is a necessary first step to determine whether the potential of video games to disseminate appropriate information is being realised. Therefore, this study aimed to investigate the frequency at which concussions were represented in four popular sports-based video games.

## Methods and materials

The gameplay of four SBVGs was analysed in an observational content analysis to explore how concussions were represented. These games were selected for their popularity and their replication of real-life athletes, tournaments, and sporting environments. Football and rugby were chosen due to their global cultural significance. Football remains the most popular sport worldwide [44], while rugby continues to grow in both participation and global reach [45]. The inclusion of games that are licensed through official governing bodies supports their relevance as meaningful case studies in evaluating how concussion is portrayed in SBVG’s. These games were EA Sports FC 25 (EAFC25), the newest release of a football game published by EA Sports (released in September 2024); EA Sports FC 24 (EAFC24, released in September 2023); Rugby League Live 4 (RLL4) a rugby league game published by Tru Blu Entertainment and Home Entertainment Suppliers in 2017; and Rugby 22 (R22), a rugby union game published by Nacon, Bigben Interactive S.A in 2022. Initially, EAFC24, RLL4 and R22 were investigated, however, EAFC25 was released during the drafting of this manuscript and therefore was included. All games were rated *“3”* on the Pan European Game Information (PEGI) scale, indicating that they are suitable for all age groups [46]. Both rugby games were the latest release of their respective series at time of analysis and all the sports had a corresponding concussion protocol published by their respective governing bodies [14,47,48].

Adherence to the STROBE (Strengthening the Reporting of Observational Studies in Epidemiology) guidelines was maintained throughout to promote transparency and maintain thorough reporting of the research process [49].

An equal amount of gameplay was analysed for each game. As the game time for rugby union and league is 80 minutes and football is 90 minutes, a common denominator of 1440 minutes of gameplay of each SBVG was analysed. This equated to 16 matches for EAFC24 and EAFC25 and 18 matches each for R22 and RLL4. During the simulation of all games default gameplay settings were applied and injury sliders (that dictate the propensity at which injuries occur) were not adjusted. Simulations for EAFC24, EAFC25 and RLL4 were conducted with both teams controlled by in-game artificial intelligence. R22 did not have this functionality, therefore one team was controlled by a researcher who was familiar with the game (IL) and the other by in-game artificial intelligence. Each game was screen-recorded using the built-in feature on the PlayStation5 (Sony; Tokyo, Japan) and were subsequently stored on a separate hard drive.

A framework to code visual and audio references to concussion and concussion protocols was developed by collating relevant definitions from pre-existing literature and adapting them for use in video games (Table 1). The resulting framework was then piloted against one match from each game by IL and the frequencies were recorded within a Microsoft Excel spreadsheet (Microsoft Corporation, USA). This pilot testing confirmed the framework’s suitability and it was implemented as per its original form during the main analyses. A random number generator was used to determine the order at which games were coded to reduce order effects. All games were coded by IL, who had considerable video analysis experience. To test interrater reliability, one match from each SBVG was selected (using a random number generator) for re-coding by FJP who was similarly experienced and was blinded to the outcomes of the initial coding. FJP’s primary research area is sport-related concussion and both FJP and IL have personal experience of the injury. IL has additional familiarity with video games, offering insight into gaming mechanics and user experience, whereas FJP approaches the subject without a gaming background, providing a contrasting perspective. Descriptive statistics were calculated using Python (Anaconda 3). Cohen’s Kappa [52] was calculated for EAFC25, EAFC24, R22 and RLL4, which returned values of *k* = 0.81, *k* = 0.93, *k* = 0.86 and *k* = 0.81 respectively. As per the thresholds described by [52], interrater reliability can be considered *‘almost perfect’* for EAFC24, *‘strong’* for EAFC25 and R22, and *‘moderate’* for RLL4. A deductive content analysis was planned to analyse any references to concussion or HIEs within in-game dialogue [53]. However, due to the absence of qualitative references to concussion in the data collected, this form of analysis was not possible and is therefore not reflected in the results.

**Table 1:** Coding framework.

Variable	Definition	Notes
Head Impact Event (HIE)	*‘A clear head impact sustained by a player […]*, *reviewed on video footage’ [*10*].*	Only direct head impacts were coded in this work, therefore the term HIEs, rather than HAEs is used. Headers, defined by Peek et al., [50]as *“an integral skill where players deliberately use their heads to re-direct the ball”*, were not included within the data set due to their intentional nature, and any HIEs associated with glitches were excluded from the data set. Only HIEs associated with a direct impact to the head were coded in this study. McLeod et al’s [10]definition of HIE was specific to rugby, but for the purposes of this study the phrase *“in the tackle”* has been removed to broaden this definition for use in football.
Commentator reference to concussion	*‘Explicit statements regarding observable concussive injuries by the sports commentators were recorded’* [51]	To be manually transcribed verbatim.
Reports of injury	Explicit reports of injury via commentator reference or dialogue boxes.	To be manually transcribed verbatim.

## Results

A total of 892 head impact events (HIEs) and 27 injuries were observed across the 68 simulated matches, representing 1440 minutes of gameplay per sport (Table 2). HIEs were caused via head-to-head, head-to-body, head-to-ground and ball-to-head mechanisms (i.e., unintentional impacts where the ball strikes the head, as opposed to deliberate headers).

**Table 2:** Head Impact Events observed during gameplay.

Video Game	Total number of HIEs observed (n)	Median number of HIEs observed per match (n, (IQR))	Total number of injuries reported during gameplay (n)	Injury diagnosis
EAFC25	331	21 (18-23)	4	3 were not specified, 1 was reported as a twisted knee by the commentators
EAFC24	253	16 (13-19)	4	Not specified
Rugby 22	215	11 (10-13)	1	Not specified
Rugby league live 4	93	5 (3-7)	18	Specified

Injuries were reported in all four games, primarily via in-game dialogue boxes and visual sequences, depending on the game. In EAFC25, four injuries were reported via a dialogue box. One of these injuries was named and referred to by a commentator as a *“twisted knee”,* one was unspecified but was reported to occur after the player was *“trodden on”,* with the remaining two injuries being unspecified and described via dialogue boxes. In EAFC24, three out of the four injuries observed were reported via dialogue boxes and referred to as *“injuries”*. Substitutes for these injuries were not made and no further detail regarding the injury type or severity was reported. The fourth injury in EAFC24 was communicated with the video game player via a video sequence where the affected player clutched his leg in pain and the referee and teammates approached during a stoppage in play. This video sequence then ended as the referee signalled for medical attention. Although no medical staff were shown in EAFC24, two separate dialogue boxes indicated that the player had been substituted and had suffered an *“injury”.* In R22 a single injury was communicated via an in-game dialogue box and referred to as a *“minor injury”*. The affected player was not substituted. In RLL4, 18 injuries were reported and named via a dialogue box: *“ankle sprain, corked thigh* (n=2)*, back muscle strain, bulging disk, neck sprain, broken arm, thigh sprain, adductor tendinopathy, concussion, rotator cuff tear, posterior cruciate ligament injury* (n=2)*, labral tear* and *hamstring”*. Overall, a greater number of HIES were observable in the football-based games, while more injuries were reported in the rugby-based games, with RLL4 being identified as having the highest number of injuries, including one instance of concussion. A single “*concussion”* was reported in the 78th minute of a RLL4 match via a dialogue box. Whilst the affected player was not observable in gameplay following this event, no notifications referenced the player being removed from play or substituted. No subgroup or sensitivity analyses were applicable within the scope of this work.

## Discussion

This study analysed four popular SBVGs and found that HIEs and injuries were observable within their gameplay. However, the representation of concussion within these SBVGs was limited to a single instance within RLL4 that was unaccompanied by any reference to, or enaction of concussion guidelines or protocols. There were no indications to suggest that the concussed player was removed from the field following the announcement of the injury. This is in contrast to the Rugby Football League Concussion Statement that states that *“Players must be removed from play if diagnosed with concussion OR if they have any signs or symptoms of concussion OR if the medical staff suspect they may have concussion” [*54*]*, highlighting a disconnect between real-life policy and in-game practice within SBVGs. The omission of real-life injury protocols is in contrast with the attention to detail in other areas, such as handshakes prior to kick-off and the accurate rendering of sponsor logos.

### A Missed Opportunity…

The absence of concussion and associated protocols within SBVGs, despite the depiction of HIEs by which a concussion could feasibly occur, can be considered a missed opportunity to promote appropriate concussion behaviours. Awareness of concussion protocols is necessary to enact them, however, literature consistently cites broad knowledge gaps within athletic populations, spanning adult, youth, female and male populations across numerous sports and countries [55–60]. Concussion management protocols are variable across sports and playing levels, however, they consistently emphasise the need to remove players with a suspected concussion from play [12,14]. This shared foundation provides a valuable opportunity for SBVGs to raise awareness of concussion management protocols.

Beyond improving player’s awareness of concussion management protocols, there is also a need to improve player’s engagement with these protocols. This need is exemplified across sport: in adolescent rugby and hockey, adherence to concussion management protocols was reported at 30.4% and across Division I and II of the National Collegiate Athletic Association, 16% of athletes reported that they had previously sustained a concussion and not reported it [61,62]. Reasons for this non-adherence to concussion management protocols included not wanting to be ruled out of matches or training, a desire to *“not let the team down”*, ignorance and peer-pressure [62,63]. There is potential that depiction of concussion management protocols in SBVGs could act to address this need. SBVGs often enable players to select digital avatars of real-life, high-profile athletes, individuals who are frequently regarded as role models [64]. These digital avatars could support the uptake of appropriate behaviours through role-model theory, where individuals are influenced by observing and imitating admired figures [65–67]. There is a need for further research to understand if role-modelling effects extend to digital versions of role models; however, this theory posits that players are more likely to adhere to guidelines if they observe role models doing the same. It must be acknowledged that the harnessing of SBVGs to raise awareness of concussion information is not a catch-all solution for the challenges associated with the portrayal of concussion in the media. Nevertheless, SBVGs are positioned to supplement existing efforts to raise awareness, particularly as they are cited as a preferred source of concussion information [17].

These findings of this study should be viewed within the broader context of harnessing the potential of SBVGs to aid the knowledge transfer of concussion management protocols. SBVGs can complement traditional methods of education given their broad audiences and high levels of user engagement. For example, people can watch SBVGs freely as streamers broadcast themselves playing in real-time to an online audience via public platforms such as Twitch (Twitch Interactive, Inc. USA). These streams are publicly accessible, available globally, and attract audiences of millions [68]. The international breadth of SBVG’s further strengthens their capacity to share concussion information. For example, EAFC25 has obtained licences from international federations, competition rights holders and individual teams to portray a broad range of professional leagues, such as the Premier League, LA LIGA, Serie A, Bundesliga, Ligue 1, and Major League Soccer, as well as UEFA [69]. Supported in 19 languages across interface, audio, and subtitles, the game is designed for accessibility and cross-cultural engagement [69]. This level of global reach provides a unique opportunity for concussion messaging to be embedded within a familiar and immersive format. Given the trust placed in such games to accurately reflect the professional game [8], the inclusion of realistic concussion protocols, such as visible player removal following suspected head injuries, could help to normalise safer behaviours. By leveraging the existing infrastructure and appeal of SBVGs, it is possible to deliver targeted health messages to a geographically and linguistically diverse audience, offering a scalable and culturally sensitive tool to support concussion education and behavioural change.

### Limitations

As the first study to examine the depiction of concussion in SBVGs, this research is exploratory in nature. While the number of analysed matches aligns with existing rugby-specific video analysis literature [70], the potential for further concussions to be observed within a larger sample of matches or video game titles cannot be ruled out. The authors acknowledge that there is a need to understand the perspectives of video game players and those who develop SBVGs to contextualise these findings and implications for future work. Collaboration with developers, medical professionals, and sporting governing bodies, teams and their sponsors is necessary to utilise the potential of SBVGs and ensure that the educational content is accurate and delivered in an ethical manner.

## Conclusion

This study found that one concussion was represented on a single occasion across a total of 68 virtual football, rugby union, and rugby league matches. This depiction was not accompanied with any reference to relevant guidelines or simulation of medical care. Enhancing the representation of concussion and its management within SBVGs could contribute to improved public understanding and encourage more appropriate responses to suspected concussions within real-world settings. Including concussion protocols within SBVGs should be viewed as part of a broader commitment within the video game industry to fulfil the social responsibility of sports teams and licence holders to raise awareness of public health issues and promote community welfare.

## Supporting information

S1 File EConcussion_Manuscript_Supplementary_Data. (XLSX)
